# Supplementary material for: OpenMM 7: Rapid development of high performance algorithms for molecular dynamics
Source: PLoS Comput Biol. 2017 Jul 26;13(7):e1005659. doi: 10.1371/journal.pcbi.1005659 (PMC5549999; doi:10.1371/journal.pcbi.1005659)
Supplement: S1 Source Code — (ZIP) [file pcbi.1005659.s002.zip › OpenMM-7.0.1-Source/docs/api-python/generated/simtk.openmm.openmm.CustomExternalForce.html]

CustomExternalForce — OpenMM 7.0.1.dev-5e86c4f documentation


# CustomExternalForce¶

*class* `simtk.openmm.openmm.``CustomExternalForce`(*\*args*)¶
:   This class implements an “external” force on particles. The force may be applied to any subset of the particles in the System. The force on each particle is specified by an arbitrary algebraic expression, which may depend on the current position of the particle as well as on arbitrary global and per-particle parameters.

    To use this class, create a CustomExternalForce object, passing an algebraic expression to the constructor that defines the potential energy of each affected particle. The expression may depend on the particle’s x, y, and z coordinates, as well as on any parameters you choose. Then call addPerParticleParameter() to define per-particle parameters, and addGlobalParameter() to define global parameters. The values of per-particle parameters are specified as part of the system definition, while values of global parameters may be modified during a simulation by calling Context::setParameter(). Finally, call addParticle() once for each particle that should be affected by the force. After a particle has been added, you can modify its parameters by calling setParticleParameters(). This will have no effect on Contexts that already exist unless you call updateParametersInContext().

    As an example, the following code creates a CustomExternalForce that attracts each particle to a target position (x0, y0, z0) via a harmonic potential:

    ```
    CustomExternalForce* force = new CustomExternalForce("k*((x-x0)^2+(y-y0)^2+(z-z0)^2)");
    ```

    This force depends on four parameters: the spring constant k and equilibrium coordinates x0, y0, and z0. The following code defines these parameters:

    ```
    force->addGlobalParameter("k", 100.0);
    force->addPerParticleParameter("x0");
    force->addPerParticleParameter("y0");
    force->addPerParticleParameter("z0");
    ```

    Special care is needed in systems that use periodic boundary conditions. In that case, each particle really represents an infinite set of particles repeating through space. The variables x, y, and z contain the coordinates of one of those periodic copies, but there is no guarantee about which. It might even change from one time step to the next. You can handle this situation by using the function periodicdistance(x1, y1, z1, x2, y2, z2), which returns the minimum distance between periodic copies of the points (x1, y1, z1) and (x2, y2, z2). For example, the force given above would be rewritten as

    ```
    CustomExternalForce* force = new CustomExternalForce("k*periodicdistance(x, y, z, x0, y0, z0)^2");
    ```

    Expressions may involve the operators + (add), - (subtract), \* (multiply), / (divide), and ^ (power), and the following functions: sqrt, exp, log, sin, cos, sec, csc, tan, cot, asin, acos, atan, sinh, cosh, tanh, erf, erfc, min, max, abs, floor, ceil, step, delta, select. All trigonometric functions are defined in radians, and log is the natural logarithm. step(x) = 0 if x is less than 0, 1 otherwise. delta(x) = 1 if x is 0, 0 otherwise. select(x,y,z) = z if x = 0, y otherwise.

    `__init__`(*self*, *energy*) → CustomExternalForce¶
    :   \_\_init\_\_(self, other) -> CustomExternalForce

        Create a CustomExternalForce.

        |  |  |
        | --- | --- |
        | Parameters: | **energy** (*string*) – an algebraic expression giving the potential energy of each particle as a function of its x, y, and z coordinates |

    Methods

    |  |  |
    | --- | --- |
    | `__init__`((self, energy) -> CustomExternalForce) | \_\_init\_\_(self, other) -> CustomExternalForce |
    | `addGlobalParameter`((self, name, ...) | Add a new global parameter that the force may depend on. |
    | `addParticle`((self, particle, parameters) -> int) | addParticle(self, particle) -> int |
    | `addPerParticleParameter`((self, name) -> int) | Add a new per-particle parameter that the force may depend on. |
    | `getEnergyFunction`((self) -> std::string const &) | Get the algebraic expression that gives the potential energy of each particle |
    | `getForceGroup`((self) -> int) | Get the force group this Force belongs to. |
    | `getGlobalParameterDefaultValue`((self, ...) | Get the default value of a global parameter. |
    | `getGlobalParameterName`((self, ...) | Get the name of a global parameter. |
    | `getNumGlobalParameters`((self) -> int) | Get the number of global parameters that the force depends on. |
    | `getNumParticles`((self) -> int) | Get the number of particles for which force field parameters have been defined. |
    | `getNumPerParticleParameters`((self) -> int) | Get the number of per-particle parameters that the force depends on |
    | `getParticleParameters`(self, index) | Get the force field parameters for a force field term. |
    | `getPerParticleParameterName`((self, ...) | Get the name of a per-particle parameter. |
    | `setEnergyFunction`(self, energy) | Set the algebraic expression that gives the potential energy of each particle |
    | `setForceGroup`(self, group) | Set the force group this Force belongs to. |
    | `setGlobalParameterDefaultValue`(self, index, ...) | Set the default value of a global parameter. |
    | `setGlobalParameterName`(self, index, name) | Set the name of a global parameter. |
    | `setParticleParameters`(self, index, particle, ...) | setParticleParameters(self, index, particle) |
    | `setPerParticleParameterName`(self, index, name) | Set the name of a per-particle parameter. |
    | `updateParametersInContext`(self, context) | Update the per-particle parameters in a Context to match those stored in this Force object. |
    | `usesPeriodicBoundaryConditions`((self) -> bool) | Returns whether or not this force makes use of periodic boundary conditions. |

    `getNumParticles`(*self*) → int¶
    :   Get the number of particles for which force field parameters have been defined.

    `getNumPerParticleParameters`(*self*) → int¶
    :   Get the number of per-particle parameters that the force depends on

    `getNumGlobalParameters`(*self*) → int¶
    :   Get the number of global parameters that the force depends on.

    `getEnergyFunction`(*self*) → std::string const &¶
    :   Get the algebraic expression that gives the potential energy of each particle

    `setEnergyFunction`(*self*, *energy*)¶
    :   Set the algebraic expression that gives the potential energy of each particle

    `addPerParticleParameter`(*self*, *name*) → int¶
    :   Add a new per-particle parameter that the force may depend on.

        |  |  |
        | --- | --- |
        | Parameters: | **name** (*string*) – the name of the parameter |
        | Returns: | the index of the parameter that was added |
        | Return type: | int |

    `getPerParticleParameterName`(*self*, *index*) → std::string const &¶
    :   Get the name of a per-particle parameter.

        |  |  |
        | --- | --- |
        | Parameters: | **index** (*int*) – the index of the parameter for which to get the name |
        | Returns: | the parameter name |
        | Return type: | string |

    `setPerParticleParameterName`(*self*, *index*, *name*)¶
    :   Set the name of a per-particle parameter.

        |  |  |
        | --- | --- |
        | Parameters: | - **index** (*int*) – the index of the parameter for which to set the name - **name** (*string*) – the name of the parameter |

    `addGlobalParameter`(*self*, *name*, *defaultValue*) → int¶
    :   Add a new global parameter that the force may depend on.

        |  |  |
        | --- | --- |
        | Parameters: | - **name** (*string*) – the name of the parameter - **defaultValue** (*double*) – the default value of the parameter |
        | Returns: | the index of the parameter that was added |
        | Return type: | int |

    `getGlobalParameterName`(*self*, *index*) → std::string const &¶
    :   Get the name of a global parameter.

        |  |  |
        | --- | --- |
        | Parameters: | **index** (*int*) – the index of the parameter for which to get the name |
        | Returns: | the parameter name |
        | Return type: | string |

    `setGlobalParameterName`(*self*, *index*, *name*)¶
    :   Set the name of a global parameter.

        |  |  |
        | --- | --- |
        | Parameters: | - **index** (*int*) – the index of the parameter for which to set the name - **name** (*string*) – the name of the parameter |

    `getGlobalParameterDefaultValue`(*self*, *index*) → double¶
    :   Get the default value of a global parameter.

        |  |  |
        | --- | --- |
        | Parameters: | **index** (*int*) – the index of the parameter for which to get the default value |
        | Returns: | the parameter default value |
        | Return type: | double |

    `setGlobalParameterDefaultValue`(*self*, *index*, *defaultValue*)¶
    :   Set the default value of a global parameter.

        |  |  |
        | --- | --- |
        | Parameters: | - **index** (*int*) – the index of the parameter for which to set the default value - **defaultValue** (*double*) – the default value of the parameter |

    `addParticle`(*self*, *particle*, *parameters*) → int¶
    :   addParticle(self, particle) -> int

        Add a particle term to the force field.

        |  |  |
        | --- | --- |
        | Parameters: | - **particle** (*int*) – the index of the particle this term is applied to - **parameters** (*vector< double >*) – the list of parameters for the new force term |
        | Returns: | the index of the particle term that was added |
        | Return type: | int |

    `getParticleParameters`(*self*, *index*)¶
    :   Get the force field parameters for a force field term.

        |  |  |
        | --- | --- |
        | Parameters: | **index** (*int*) – the index of the particle term for which to get parameters |
        | Returns: | - **particle** (*int*) –   the index of the particle this term is applied to - **parameters** (*vector< double >*) –   the list of parameters for the force field term |

    `setParticleParameters`(*self*, *index*, *particle*, *parameters*)¶
    :   setParticleParameters(self, index, particle)

        Set the force field parameters for a force field term.

        |  |  |
        | --- | --- |
        | Parameters: | - **index** (*int*) – the index of the particle term for which to set parameters - **particle** (*int*) – the index of the particle this term is applied to - **parameters** (*vector< double >*) – the list of parameters for the force field term |

    `updateParametersInContext`(*self*, *context*)¶
    :   Update the per-particle parameters in a Context to match those stored in this Force object. This method provides an efficient method to update certain parameters in an existing Context without needing to reinitialize it. Simply call setParticleParameters() to modify this object’s parameters, then call updateParametersInContext() to copy them over to the Context.

        This method has several limitations. The only information it updates is the values of per-particle parameters. All other aspects of the Force (such as the energy function) are unaffected and can only be changed by reinitializing the Context. Also, this method cannot be used to add new particles, only to change the parameters of existing ones.

    `usesPeriodicBoundaryConditions`(*self*) → bool¶
    :   Returns whether or not this force makes use of periodic boundary conditions.

        |  |  |
        | --- | --- |
        | Returns: | false |
        | Return type: | bool |

    `__delattr__`¶
    :   x.\_\_delattr\_\_(‘name’) <==> del x.name

    `__format__`()¶
    :   default object formatter

    `__getattribute__`¶
    :   x.\_\_getattribute\_\_(‘name’) <==> x.name

    `__hash__`¶

    `__reduce__`()¶
    :   helper for pickle

    `__reduce_ex__`()¶
    :   helper for pickle

    `__sizeof__`() → int¶
    :   size of object in memory, in bytes

    `__str__`¶

    `getForceGroup`(*self*) → int¶
    :   Get the force group this Force belongs to.

    `setForceGroup`(*self*, *group*)¶
    :   Set the force group this Force belongs to.

        |  |  |
        | --- | --- |
        | Parameters: | **group** (*int*) – the group index. Legal values are between 0 and 31 (inclusive). |

High performance molecular simulation on GPUs


### Navigation

- Application Layer
- Library Layer
  - Core Objects
  - Forces
  - Integrators
  - Extras

©2015, Stanford University and the Authors.
|
Powered by Sphinx 1.3.1
& Alabaster 0.7.7
|
Page source
